# Antiphospholipid Syndrome and Preeclampsia in Pregnancy: A Case Report

**DOI:** 10.7759/cureus.28458

**Published:** 2022-08-27

**Authors:** Rafailia Skoura, Paraskevi-Eva Andronikidi, Doxakis Anestakis, Savvas Petanidis, Eirini Orovou, Maria Tzitiridou, Panagiotis Eskitzis

**Affiliations:** 1 Midwifery, University of Western Macedonia, Ptolemaida, GRC; 2 Medicine, University of Crete, Crete, GRC; 3 Histology, University of Nicosia, Nicosia, CYP; 4 Medicine, Aristotle University of Thessaloniki, Thessaloniki, GRC

**Keywords:** antiphospholipid antibodies, pregnancy, autoimmune disease, preeclampsia, antiphospholipid syndrome

## Abstract

Antiphospholipid syndrome (APS) refers to a clinical autoimmune syndrome characterized by arterial or venous thrombosis and pregnancy morbidities, such as fetal loss after the 10th week of gestation, recurrent miscarriages, or intrauterine growth restriction. This study describes a case of preeclampsia in a 37-year-old primiparous woman in the 30th week of pregnancy with a lack of prior thrombotic history. The birth of a dead neonate and the findings of placenta thrombosis raised the suspicion of APS, which was confirmed by the finding of antibodies. A description of the treatment, which is still under investigation, follows. In our case, tissue sections were stained followed by observation. Various placental changes were detected with the presence of placental intravascular thrombi. The most important finding of this case study is the presence of severe preeclampsia in the setting of APS, with no previous medical history. In conclusion, antiphospholipid syndrome can be directly related to preeclampsia during pregnancy, leading to complications that may be preventable if immediate medical intervention is available.

## Introduction

Antiphospholipid syndrome (APS) is an autoimmune disease with increased production of antiphospholipid antibodies (aPL) and represents an acquired form of thrombophilia [1]. Additionally, obstetric events are likely to occur. In case of a thrombotic event, such as clot formation in a vein or artery, the APS is renamed “thrombotic antiphospholipid syndrome”, while in case of an obstetric event, such as miscarriage, premature ectopic pregnancy, fetal death, delayed fetal development, preeclampsia, etc., it is called “obstetric antiphospholipid syndrome” [1,2]. It is defined as primary when it occurs without a medical history of other diseases, and secondary when there is another autoimmune disease present, such as systemic erythematosus lupus (SEL). Finally, it is called catastrophic when it involves organ failure [1-3]. The diagnosis of APS is based on the detection of aPL. More specifically, APS requires at least one clinical and laboratory criterion to be met for diagnosis. Lupus or anticardiolipin anticoagulants are found in more than two samples at a medium or high level of IgG-IgM antibodies over a period of at least 6 weeks [4]. Furthermore, aPL should be tested in patients under the age of 50 with a thrombotic episode and in women with a history of obstetric complications, such as fetal loss after the 10th week of gestation, recurrent miscarriages, or intrauterine growth restriction (IUGR), as they are more likely to have APS [1]. Several studies in rheumatology and hematology patients have evaluated that anti-β2 glycoprotein I (anti-β2-GPI) may be more specific than anticardiolipin (aCL)antibodies for the clinical APS diagnosis [5]. The incidence of APS in pregnancy ranges from 0 to 11% (with an average of 2%). However, the APS is detected in up to 37% of cases with SEL [6].

Over the last 30 years, there has been ongoing research on APS management. Recent studies have shown that low-dose aspirin (75-100 mg/day) is recommended for people at high risk, as well as for patients with APS. The role of aspirin in prevention and risk reduction was confirmed by studies. Finally, it should be noted that treatment with hydroxychloroquine has helped to reduce the risk of patients developing APS [7]. 

Preeclampsia is a pregnancy disorder and a warning sign that APS and more specifically, obstetric antiphospholipid syndrome, may develop. Preeclampsia involves hypertension starting at 20 weeks of pregnancy, as well as proteinuria (although not always present) in most cases. However, antihypertensive therapy generally should be started after the confirmation of severe hypertension, in which case, the criteria for severely increased blood pressure can be met without waiting until 4 hours to pass. Proteinuria is defined as ≥300 mg per 24-hour urine collection (or this amount extrapolated from a timed collection) or protein:creatinine ratio ≥0.3, or urine dipstick reading ≥2+ (if other quantitative methods are not available). In the absence of proteinuria, new arrival of high blood pressure with the new arrival of any of the following: renal insufficiency (serum creatinine of >1.1 mg/dL [97 micromol/L] or doubling of the serum creatinine concentration in the absence of other renal diseases) or thrombocytopenia (platelet count <100,000/microL), decreased liver function as indicated by liver transaminase levels at least twice the usual centralization, persistent visual or cerebral symptoms, as well as pulmonary edema [8, 9].

Furthermore, the 2016 American College of Obstetricians and Gynecologists guidelines indicated that low-dose aspirin (81 mg/day) is effective in preventing preeclampsia in pregnant women with hypertension [10] and research to date has shown there is a significant change in the outcome of pregnancies that have followed that guideline [10]. It has also been shown that aspirin is the only drug for which there is some positive evidence of preventing preeclampsia. Another drug that has been used in cases of preeclampsia is metformin, which, nevertheless, has been tested in random cases and has not shown any strong changes to make it widely known [11]. Treatment of preeclampsia is based on the completion of childbirth. It is recommended that labor should be performed in the 37th - 38th week of pregnancy [12]. Nevertheless, the time of delivery depends on the severity of the symptoms [13].

The correlation of aPL with preeclampsia and other negative pregnancy outcomes, such as IUGR, was first described in the early 1980s. Several studies found that women with APS have an increased risk of severe preeclampsia. Nevertheless, as preeclampsia usually occurs in 4% to 6% of pregnant women in the second semester, only a small proportion of women with this condition, and an absence of other risk factors, is tested for APS [14].

The importance of this case study is to emphasize the rarity of the event and to show the risk factors for its occurrence in pregnancy, but also to take preventive measures for successful pregnancy outcomes.

## Case presentation

A 37-year-old Caucasian nulliparous woman in the 30th week of pregnancy visited the maternity hospital with high blood pressure (180/110 mmHg) and visual disturbances and headache. The ultrasound examination showed an absence of fetal pulses, and the woman was hospitalized for further monitoring and delivery. The woman's previous examination was at 26 weeks, during which no abnormal blood tests, abnormal blood pressure values, or abnormal ultrasound imaging of the fetus were observed. Intravenous dihydralazine was immediately administered by the nephrologist on duty to lower the blood pressure, while the woman was prepared for an emergency cesarean section. She was not a smoker, did not take aspirin, and had a free medical history and an unknown family history of thrombophilic disorder.

After delivery, the placenta of the dead IUGR fetus was sent for histologic evaluation. The laboratory blood serum tests requested by the nephrologist, rheumatologist, and obstetricians of the postpartum woman, showed that the routine analyses were within the normal range, but high anti-β2GPI domain I anti-body titles were found. Anti-beta2 glycoprotein I antibody of IgG was found in serum present >40U/mlon on two occasions (45U/ml and 42U/ml). The first value was right after the delivery and the second 15 weeks later. The antibodies were measured by a standardized enzyme-linked immunosorbent assay (ELISA). Furthermore, ELISA was found to be negative for anticardiolipin (aCL) antibodies (immunoglobulin IgG and IgM) as was the lupus anticoagulant (LA) assay. Clotting tests: partial thromboplastin time (PTT), prothrombin time (PT), activated partial thromboplastin clotting time​​​​​​​ (​​​​​​​aPTT), and platelet count were found to be in normal values [15]. However, during her hospitalization, an upper respiratory tract infection unrelated to COVID-19 was observed.

**Figure 1:**
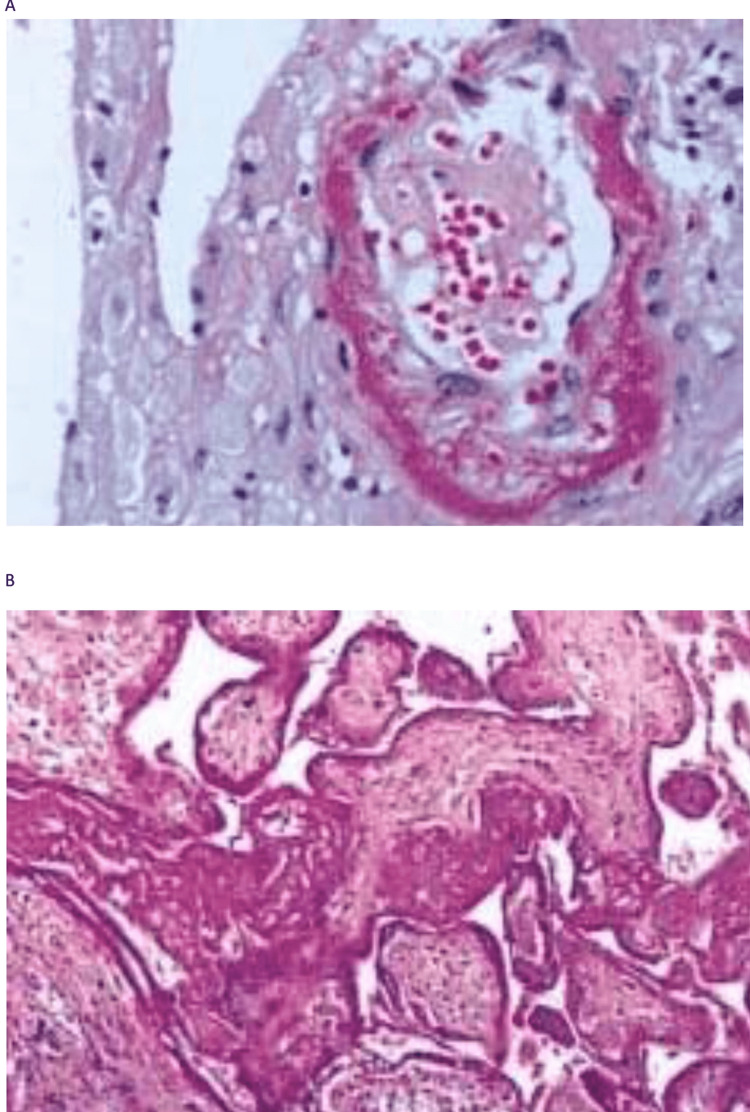
A placenta associated with preeclampsia showing decidual placental vasculopathy (A) and placental villous infarcts (B) A: A decidual vessel shows fibrinoid necrosis of vessel walls and a perivascular mononuclear infiltrate. B: Infracted areas show the aggregation of ghost-like necrotic villi.

The eosin and hematoxylin dying technique was used for the tissue sections (Figure 1). The protocol of the procedure was: 1) deparaffinized the part: flame the slide on the burner and place in the xylene and repeat the treatment, 2) moisturizing: hydrate the tissue section by passing through abatement centralization of alcohol baths and water from 100% to 70%, 3) stain in hematoxylin for 4 minutes, 4) wash in running tap water until sections turn “blue” for up to 5 minutes, 5) modify in 1% acid alcohol (1% hydrochloric acid​​​​​​​ [HCl]** **in 70% alcohol) for 1 second, 6) wash in running tap water, 7) stain in eosin for 2 minutes, 8) drying in raising the concentration of alcohols and clean in xylene, 9) put on mounting media.

After the staining process, significant results were obtained from the examined sections under the microscope. The placenta was evaluated and the microscopic findings included acute inflammatory and non-inflammatory changes of amnion, infarctions, intervillous thrombosis, chorionic villitis, hemorrhagic endovasculitis, placental intravascular thrombi, trophoblast degenerative knots, perivillous fibrin deposition and fibrinoid necrosis, erythroblastosis and villous oedema.

## Discussion

APS is responsible for venous thrombosis and adverse effects on pregnancy (recurrent pregnancy loss or IUGR syndrome) and the presence of aPL is necessary [1]. However, the APS in pregnancy may be associated with SEL, but occasionally with preeclampsia [8]. In our patient, there are vascular, pregnancy criteria (preeclampsia), and laboratory tests (positive values for the anti-β2GPI domain I antibodies). Therefore, this is a case of obstetric antiphospholipid syndrome, which presents with severe preeclampsia and thrombosis [1,2].

As previously mentioned, preeclampsia is directly related to APS and in particular to anti-β2GPI domain I. Preeclampsia is an emergency situation in pregnancy that can affect both mother and fetus through serious complications [12]. However, during pregnancy, normal changes occur in the coagulation system of a woman. A reduction in free and total S protein and also a raise in coagulation factors Vc, VIIIc, Xc, and von Willebrand factor antigen are observed [6]. As we saw in our 30-week gestation case, fetal death appears to have resulted from massive placental thrombosis, while the mechanisms associated with preeclampsia are unknown. For this reason, preeclampsia was not considered a main criterion of APS, but its presence can be a favorable factor for the development of APS with a rate of approximately 18% [16]. It has been proven that severe preeclampsia is mainly associated with the presence of anti-β2GP1. Therefore, women with severe preeclampsia should be screened for the possibility of APS to prevent future complications in pregnancy [17, 18].

This case study shows the impact of untimely diagnosis and highlights the importance of multidisciplinary management of this type of pregnancy complication in order to avoid further complications, safer obstetric care, and desired outcomes.

## Conclusions

In conclusion, this case study described a combination of preeclampsia and APS in a pregnant woman at 30 weeks. Although one clinical criterion is required for the diagnosis of antiphospholipid syndrome, at least three conditions related to the syndrome were there in our presentation, including severe preeclampsia. This case study also shows the importance of interdisciplinary collaboration (obstetrics, nephrology, and rheumatology) with the aim of achieving proper care for pregnant women and preventing possible complications. The sudden onset of preeclampsia and the lack of prior thrombotic history left no possibility of screening for APS. However, the adverse outcomes of the present pregnancy signal the suspicion of comorbidity.
